# Novel PVDF-PEG-CaCO_3_ Membranes to Achieve the Objectives of the Water Circular Economy by Removing Pharmaceuticals from the Aquatic Environment

**DOI:** 10.3390/membranes13010044

**Published:** 2022-12-29

**Authors:** Maciej Szwast, Daniel Polak, Wiktoria Arciszewska, Izabela Zielińska

**Affiliations:** Faculty of Chemical and Process Engineering, Warsaw University of Technology, Warynskiego 1, 00-645 Warsaw, Poland

**Keywords:** membrane, pharmaceutical removal, circular economy, water, aquatic environment

## Abstract

In the aquatic environment, substances of pharmacological origin are common contaminants. The difficulty of removing them from water is a problem for the implementation of a circular economy policy. When recycling water, an effort should be made to remove, or at least, minimize the presence of these substances in the water. Porous membranes with a new functionality consisting in their adsorption capacity towards pharmaceutical substances have been developed. A Polyvinylidene Fluoride (PVDF) membrane with Calcium Carbonate (CaCO_3_) nanoparticles as an adsorbent was prepared. By implementing an integrated filtration-adsorption process using sulphadiazine, as a representative of pharmacological substances, 57 mg/m^2^ of adsorption capacity has been obtained, which is an improvement in adsorption properties of more than 50 times that of a commercial membrane. At the same time the membrane permeability is 0.29 m^3^/(h·m^2^·bar), which means that the membrane’s permeability was improved by 75%.

## 1. Introduction

Due to the needs of the natural environment and tightening regulations, the principles of the circular economy concept are being implemented in more and more new areas of industry [1,2,3]. The main idea behind this concept is to reuse as much as possible of all the substrates and products put into circulation. This is considered, for example, in the context of substances such as plastics [4], electro-waste [5], minerals [6], nutrients [7], or products of the pharmaceutical and cosmetics industries [8,9]. However, there is a strong emphasis on the recovery of water, particularly potable water, used for agricultural, domestic, and industrial purposes [10,11,12,13]. All activities that aim to purify water of substances contained in it, and to return the water for reuse in the same or other processes, serve the objectives of a circular economy. Processes such as filtration, adsorption, flotation, coagulation, sedimentation, precipitation, or biological methods have been used to treat water for many years [14,15,16,17]. Additionally, photocatalytic methods can be successfully used [18,19,20].

Membrane processes, in particular techniques in which pressure is the driving force, deserve special attention when it comes to water treatment and water cycle closure [21,22,23]. In processes such as microfiltration, ultrafiltration, nanofiltration, and reverse osmosis, it is possible to separate suspended particles, colloids, multivalent, and monovalent salts from water [24,25]. In contrast, other membrane techniques such as pervaporation allow the separation of organic solvents or volatile compounds from water [26,27]. Membrane processes can fulfil two purposes: the treatment of water and the recovery of the constituents in the water and their reuse. It is this approach to the implementation of membrane processes that meets the basic tenets of a circular economy [28,29]. In recent years, there has been growing interest in the use of integrated processes, including membrane processes. By integrating with processes such as adsorption, absorption, or crystallization, a synergy of the properties of the individual processes can be achieved [30,31,32]. To implement an integrated membrane process, therefore, a membrane should be used that allows the filtration process and the other processes, with which it is integrated to, run simultaneously. The authors of this paper have developed a range of membranes that integrate filtration and adsorption processes [33,34].

An important issue in a circular economy is the return of freshwater for reuse, suitable for domestic and agricultural purposes. An important and still not fully resolved problem is the presence of products of pharmaceutical origin in water [35]. The problem of pharmaceuticals in agricultural and anthropogenic wastewater has been growing. This is due to the growing use of antibiotics and anti-inflammatory drugs, which are increasingly available without a doctor’s prescription. As only a proportion of drugs are metabolised by humans and animals, a large proportion of these substances enter the water cycle unchanged. Adverse phenomena such as the poisoning of aquatic organisms and the resistance to antibiotics of certain bacterial strains are associated with the presence of pharmaceuticals, which are not removed from the water by classical water treatment processes. The processes involved in closing water cycles must therefore take into account the need to remove pharmaceutical substances.

The aim of this study is to develop a new membrane that, by implementing an integrated process of water filtration and pharmaceutical adsorption, will enable the achievement of a circular economy.

## 2. Materials and Methods

### 2.1. Membranes and Their Modification

A19-series tubular membranes made of Polyvinylidene fluoride (PVDF) with a cut-off value of 20 kDa (PCI Membranes, UK) were used in the tests.

These membranes were then modified by dip-coating (20 mm/s of withdrawal velocity, conducted in room temperature) with appropriately prepared solutions. The base of these solutions was a 5% wt. solution of PVDF (Sigma Aldrich, Poznan, Poland) (ca. 534 kDa) in N-methyl pyrrolidone (NMP) (Sigma Aldrich, Poznan, Poland) (ACS reagent, ≥99.0%). Poly(ethylene glycol) (PEG 200) (Sigma Aldrich, Poznan, Poland) (purity: for synthesis) was added to this solution in amounts corresponding to 10% wt., 15% wt., and 20% wt. of PVDF weight, respectively. PVDF + PEG solutions were also prepared, to which CaCO_3_ nanoparticles were added in amounts corresponding to 2% wt., 5% wt., and 10% wt. of the PVDF weight, respectively. Nanoparticles were produced in-house in a disk reactor, according to the technology presented in the paper [36], using calcium hydroxide (Chempur, Piekary Slaskie, Poland) (analytically pure) and carbon dioxide (99.99%, Multax, Zielonki-Parcela, Poland) as substrates. The average size of the nanoparticles obtained was 50 nm. The size of particles measured were taken from paper [36] and was confirmed by our own measures by Scanning Electron Microscopy (SEM).

Compositions of the modification solutions of the membranes tested are shown in Table 1.

The cylindrical membranes that were modified are of composite construction. On the inside of the tube there is a selective PVDF layer, while on the outside there is a fibrous support layer. During the tests, being the subject matter of this paper, the outer side of the cylindrical membrane was modified.

### 2.2. Analysis of Physicochemical Properties

The membranes were characterised using chemometric methods to gather information on their morphology and physicochemical properties.

Scanning electron microscopy allows to assess the uniformity of the membrane coating with the modification layer. It is also possible to assess the uniformity of the dispersion of CaCO_3_ nanoparticles across the entire membrane surface. The PhenomPro microscope (PhenomWorld, Eindhoven, the Netherlands) was used in the tests.

Measurements of membrane surface tension allow to assess the hydrophilicity of membranes. For water filtration processes, hydrophilic membranes are advisable. The tests carried out allowed for the assessment of the impact of membrane modifications on their surface properties. In the tests, the sessile drop method was used. A 0.5 µm droplet of demineralised water was deposited on the membrane. The OCA 25 device was used (DataPhysics Instruments, Filderstadt, Germany).

The pore size distribution in the unmodified membrane and in the modified membranes was studied using the flow porosimetry method. The Capillary Flow Porometer iPore −1200 A device (PMI, Ithaca, NY, USA) was used in the tests.

### 2.3. Testing of Process Properties

The fabricated membranes were incorporated into membrane modules. Four membranes with an active length of 45 cm were placed in each membrane module, which, at a membrane diameter of 12.5 mm, gave a membrane area of 0.07 m^2^ per membrane module.

The module was placed in a custom-built laboratory test facility consisting of a feed tank, a permeate tank, a pump, flow and pressure gauges, and valves. This installation allows the measurement of the membrane permeability and to analyze the micro-/ultrafiltration process.

Membrane permeability tests were performed using demineralised water. Permeate flux was measured at a constant feed pressure of 1 bar. The membrane permeability *P* was determined from the following relationship:

(1) $$P=\frac{Q_{p}}{S\cdot p_{TM}}$$

where *Q_p_* is the permeate flux volume, *S* is the surface area of the membrane being tested, and *p_TM_* is the transmembrane pressure.

Tests of the integrated process, i.e., the filtration-adsorption process, were carried out using a sulfadiazine solution as a substance representing pharmaceutical compounds. Solutions of 40 mg/L were prepared in demineralized water. During the process, the retentate and permeate streams were returned to the feed tank, ensuring that the process could run for a long time with a finite volume of solution. Liquid samples taken at various moments during the process were subjected to spectrophotometric tests, to determine the current concentration of the pharmaceutical substance in the liquid. The Gensys 10S UV-Vis instrument (ThermoFisher Scientific, Waltham, MA, USA) was used in the measurements. For measurement purposes, a calibration curve was previously made for the tested solution.

By analysing the adsorption capacity of the membranes, the mass of the adsorbed component per unit membrane area was calculated. The following relationship was used for this purpose:

(2) $$q=\frac{c_{t}-c_{t-1}}{S}\cdot V$$

where *c_t_* is the concentration of the substance measured at time *t*, *c_t_*_−1_ is the concentration of the substance measured at the previous time point, *S* is the surface area of the membrane, and *V* is the volume of solution circulating through the system.

## 3. Results and Discussion

### 3.1. Water Permeability Tests

**Figure 1:**
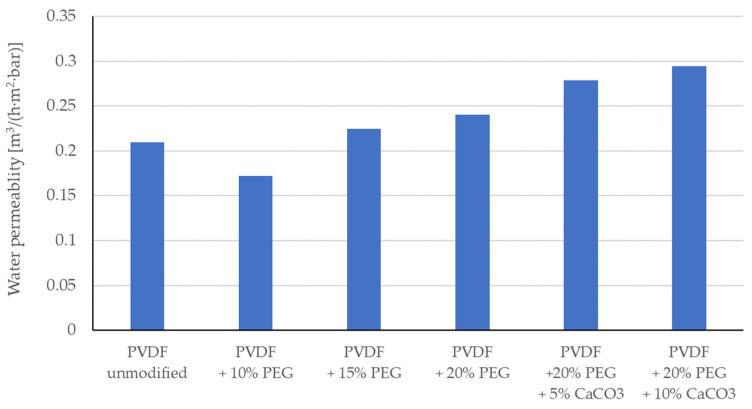
Water permeability results for the membranes tested.

The first test, to which the membranes were subjected to, was the measurement of the ultrapure water permeability coefficient for membranes modified only with a solution consisting of N-methyl pyrrolidone (NPM) solvent, PVDF polymers (5% wt.), and PEG polymers (10% wt., 15% wt. and 20% wt.). The aim of these measurements was to test the effect of the presence of PEG on membrane permeability. According to literature reports, the presence of PEG affects the porosity and permeability of membranes made of PVDF [37,38]. The results, Figure 1, show that at lower PEG concentrations the membrane permeability decreases, while at higher concentrations the membrane permeability is higher than for the unmodified membrane. Therefore, the decision was made to carry out further modifications with solutions (suspensions) containing CaCO_3_, using solutions of 20% wt. PEG. Further permeability measurements were carried out for membranes modified with suspensions made based on NMP + 5% wt. solution, PVDF + 20% wt., and PEG + CaCO_3_ (5% wt. or 10% wt.). The results of permeability measurements for such membranes are also summarised in Figure 1. It should be noted that for higher concentrations of CaCO_3_, higher membrane permeability was obtained than at lower concentrations. This is probably due to the increased hydrophilicity of the membrane [39,40], but SEM and porosimetry analysis were also done and presented in this paper. The increased hydrophilicity of the membranes is confirmed by the results of the contact angle measurements presented later in this paper (Table 2).

Membranes with the following markings were therefore further tested and analysed: PVDF unmodified, PVDF + 20% PEG, PVDF + 20% PEG + 5% CaCO_3_, and PVDF + 20% PEG + 10% CaCO_3_.

### 3.2. Goniometric Test

The membranes selected for further testing were subjected to contact angle tests. These measurements were made to control changes in membrane hydrophilicity. Hydrophilic membranes are recommended for the implementation of water purification processes. The tests discussed in this paper aimed to check the direction of change of this parameter, as a result of the modifications carried out. Contact angle values were measured for both the inner and outer layers of the cylindrical membrane. The results of the measurements are summarised in Table 2.

Analysis of the contact angle measurement data allows the conclusion that the presence of both PEG and CaCO_3_ nanoparticles in the membrane-modifying solution (suspension) leads to a decrease in the contact angle value, i.e., an increase in the hydrophilicity of the membrane. Such changes are visible on both the outer (modified) side of the cylindrical membrane and on the inner side. This leads to the further observation that the modification carried out on the outer side also affected the inner side of the membrane, i.e., the side where the actual selective layer of the unmodified membrane is located.

The observed increase in hydrophilicity of the modified membranes is reflected in the increased permeability of the membranes to ultrapure water, Figure 1.

### 3.3. SEM Analysis

**Figure 2:**
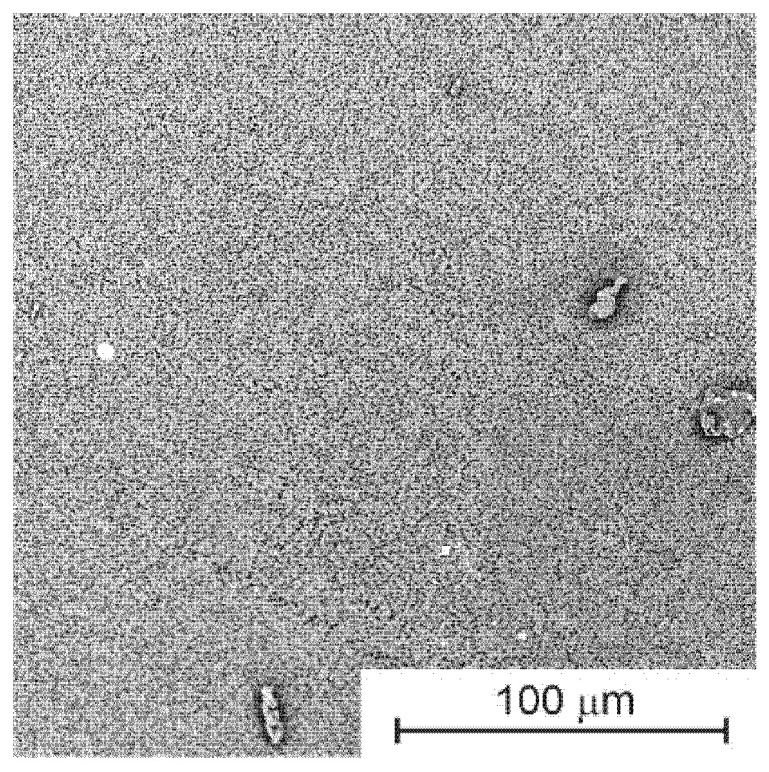
SEM image of the inside of a cylindrical membrane.

The results of the contact angle measurements presented above suggest that the modifications led to changes in the structure of both the outer layer and the inner layer of the cylindrical membrane. Observations of SEM images of the inner layer do not allow such changes to be confirmed unequivocally. These changes are therefore too subtle to be imperceptible in microscopic observations. An example SEM image of the inner side of a cylindrical membrane is shown in Figure 2.

**Figure 3:**
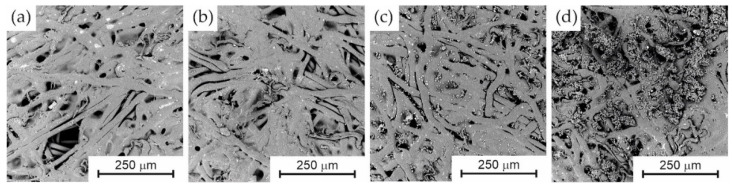
SEM images of the outer side of cylindrical membranes for different modifications. (**a**) PVDF unmodified, (**b**) PVDF + 20% PEG, (**c**) PVDF + 20% PEG + 5% CaCO_3_, and (**d**) PVDF + 20% PEG + 10% CaCO_3_.

However, the modifications carried out resulted in changes observable on SEM images, occurring on the outer side of the cylindrical membrane. Figure 3 presents images showing the exterior of the modified membrane for different modifications.

Analysis of Figure 3 allows the following observations. Modification of the membrane with PVDF and PEG solution does not produce noticeable changes in the structure of the outer side of the cylindrical membrane, Figure 3a,b. No new polymer bridges are seen to appear between the fibres that make up the structure of the membrane’s outer layer. The increased hydrophilicity of the modified membrane may be due to the coating of the fibres with an ultra-thin polymer layer, which cannot be detected by this type of microscope. In contrast, Figure 3c,d show CaCO_3_ nanoparticles in the form of larger or smaller agglomerates. The agglomeration of nanoparticles is obviously an unfavourable phenomenon from the point of view of the development of the active surface, however, the distribution of this compound on the membrane surface appears to be quite uniform. For a membrane containing a higher concentration of CaCO_3_, the number of observed nanoparticles on the membrane surface is significantly higher.

### 3.4. Porosimetry Analysis

**Figure 4:**
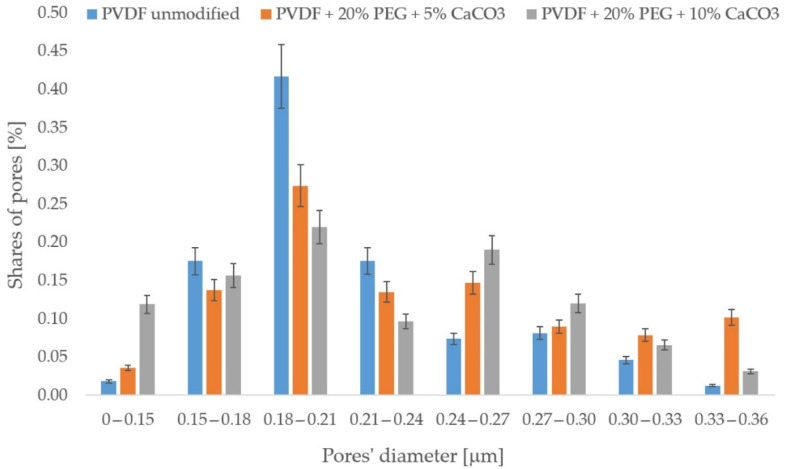
Results of porosimetry tests.

In order to investigate the effect of the modification on the structure of the porous membrane, porosimetry tests were performed. The membranes tested were PVDF unmodified, PVDF + 20% PEG + 5% CaCO_3,_ and PVDF + 20% PEG + 10% CaCO_3_. The results of the measurements are shown in Figure 4.

Analysis of Figure 4 shows that the modifications carried out do not significantly affect the porosity of the membranes. No sealing of existing pores or destruction of the porous structure by the solvent in the modifying solution is observed. It can therefore be concluded that the increased permeability of the modified membranes is due to changes in the membrane surface properties, as evidenced by measurements of the contact angle of the membrane surfaces.

### 3.5. Process Tests

As part of the process tests, measurements were taken of the change in permeate flux over time for the different types of membranes prepared, and the mass of the adsorbed pharmaceutical substance, i.e., sulphadiazine, according to Equation (2).

**Figure 5:**
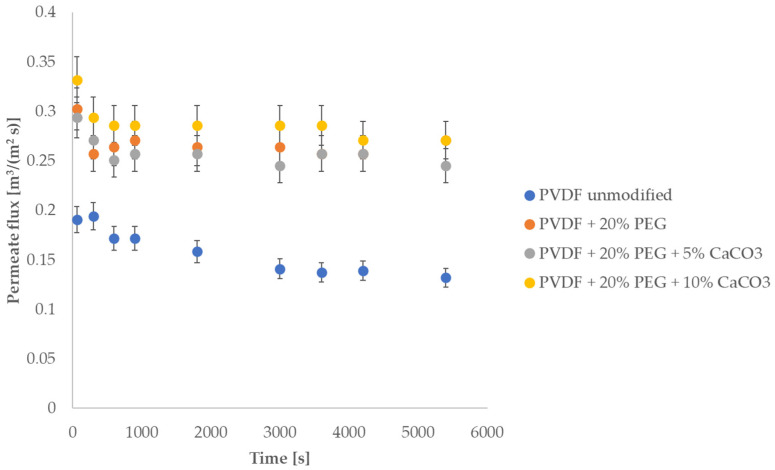
Change in permeate flux when running the process with sulfadiazine.

Figure 5 shows the change in permeate flux for the different membranes over time during the integrated filtration-adsorption process.

Tests performed in a closed loop system indicate fairly typical changes in permeate flux over time. The permeate flux decreases slightly over time. There is no noticeable effect of fouling or significant loss of membrane filtration properties. It should be noted, however, that the filtrate flux is highest for membranes with the lowest contact angle value (Table 2), while it is lowest for membranes with the highest contact angle value. Such relationship between the value of the contact angle and the value of the permeate flux was expected [40,41].

**Figure 6:**
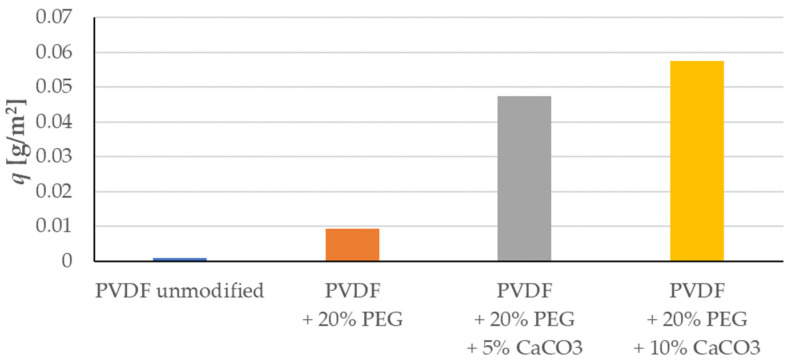
Mass of adsorbed sulfadiazine in the process (after 90 min).

The change in concentration of the pharmaceutical substance in the filtered solution was also measured during the process, allowing the change in mass adsorbed on the membrane during the process to be determined. The results of the measurements are shown in Figure 6.

Analysis of Figure 6 shows a very clear positive effect of the presence of CaCO_3_ nanoparticles on the adsorption properties of the membrane. The unmodified PVDF membrane has virtually no adsorption capacity. Covering this membrane with a PEG solution does not bring about fundamental changes in the adsorption properties. In turn, membrane modification with suspensions containing CaCO_3_ nanoparticles significantly improves the membrane’s adsorption properties for pharmacological agents, represented here by sulfadiazine. A twofold increase in the concentration of CaCO_3_ nanoparticles does not yield a twofold improvement in adsorption properties. This increase is 20%. However, it is a significant enough increase to justify the use of more nanoparticles in the membrane fabrication process.

The membranes obtained in this work are characterised by better adsorption properties and a higher permeability value than membranes made of Cellulose Acetate/P4VP-b-PEO and presented in [42]. Comparing the results of our paper with the results of papers [43,44] concerning the integrated ultrafiltration-photocatalysis process, it can be concluded that the degree of sulfadiazine removal is comparable for both methods with similar permeability values. The advantage of the proposal of this work is the integration of processes in one membrane module without the need to use external energy, as is the case of photocatalysis.

## 4. Conclusions

A new type of membrane has been developed that may find application in the purification of aqueous environments from pharmacological contaminants. The use of this type of membrane is part of the policy of a circular economy. Thanks to these membranes, it is possible to close off the circuits of water streams and reuse them not only for industrial purposes, but also for food purposes.

The developed membranes are characterised by increased permeability compared to commercially available membranes, they have unchanged selectivity characteristics, and their properties allow adsorption, with significant amounts of pharmacological agents.

## Figures and Tables

**Table 1:** Compositions of membrane modification solutions.

Membrane Marking	PVDF	PEG	CaCO_3_
PVDF unmodified	0% wt.	0% wt.	0% wt.
PVDF + 10% PEG	5% wt.	10% wt.	0% wt.
PVDF + 15% PEG	5% wt.	15% wt.	0% wt.
PVDF + 20% PEG	5% wt.	20% wt.	0% wt.
PVDF + 20% PEG + 5% CaCO_3_	5% wt.	20% wt.	5% wt.
PVDF + 20% PEG + 10% CaCO_3_	5% wt.	20% wt.	10% wt.

**Table 2:** Contact angles of the inner and outer surfaces of membranes.

Membrane Marking	Contact Angle	
	Internal Surface	External Surface
PVDF unmodified	105.2°	82.6°
PVDF + 20% PEG	96.4°	80.2°
PVDF + 20% PEG + 5% CaCO_3_	90.4°	78.3°
PVDF + 20% PEG + 10% CaCO_3_	93.0°	71.3°

## Data Availability

Not applicable.
